# Therapeutic targeting of microglia mediated oxidative stress after neurotrauma

**DOI:** 10.3389/fmed.2022.1034692

**Published:** 2022-11-03

**Authors:** Austin N. Smith, Michael Shaughness, Sean Collier, Deanna Hopkins, Kimberly R. Byrnes

**Affiliations:** ^1^Neuroscience Program, Uniformed Services University of the Health Sciences, Bethesda, MD, United States; ^2^Henry M. Jackson Foundation for the Advancement of Military Medicine, Inc., Bethesda, MD, United States; ^3^Department of Anatomy, Physiology and Genetics, Uniformed Services University of the Health Sciences, Bethesda, MD, United States

**Keywords:** iron, microglia, mitochondria, NADPH oxidase, oxidative stress, spinal cord injury, traumatic brain injury

## Abstract

Inflammation is a primary component of the central nervous system injury response. Traumatic brain and spinal cord injury are characterized by a pronounced microglial response to damage, including alterations in microglial morphology and increased production of reactive oxygen species (ROS). The acute activity of microglia may be beneficial to recovery, but continued inflammation and ROS production is deleterious to the health and function of other cells. Microglial nicotinamide adenine dinucleotide phosphate (NADPH) oxidase (NOX), mitochondria, and changes in iron levels are three of the most common sources of ROS. All three play a significant role in post-traumatic brain and spinal cord injury ROS production and the resultant oxidative stress. This review will evaluate the current state of therapeutics used to target these avenues of microglia-mediated oxidative stress after injury and suggest avenues for future research.

## Introduction

Traumatic injury to the central nervous system (CNS), including the brain (traumatic brain injury, TBI) and spinal cord (SCI), affects millions of people every year, adding to those already living with these injuries (1–3). Microglia are the brain’s primary immunocompetent cells; these cells’ response to CNS trauma ranges from restorative to detrimental and studies have shown microglial activity after a traumatic event shapes recovery and impacts functional and behavioral outcomes (4, 5). Post-injury phagocytosis, neurotrophic support, and release of mediators of cytotoxicity, including reactive oxygen species (ROS), fall into the category of microglial responses (6). In this review, we collect recent efforts to use pharmacological therapies to mediate microglial production of ROS in the CNS after trauma to improve functional and behavioral outcomes.

The vast functions of neurons and glia require high energy expenditure that is dependent upon oxygen for mitochondrial ATP production. However, as an electron acceptor, oxygen can form ROS. ROS include but are not limited to superoxide (O_2_^–^), peroxide (O_2_^2–^), and hydroxyl radical (•OH). While low levels of ROS act as signaling molecules, high levels of ROS can cause cellular damage. To maintain homeostasis, cells increase antioxidants to offset accumulated ROS.

**FIGURE 1:**
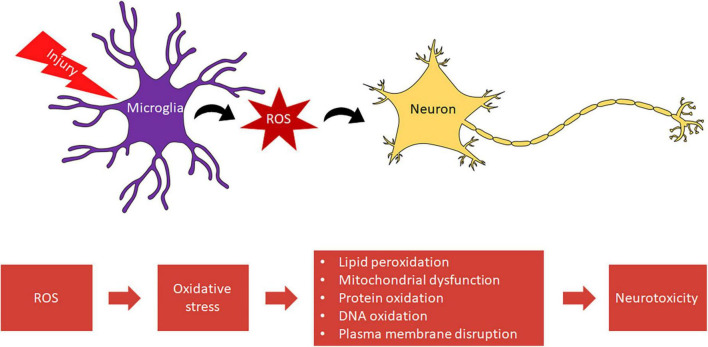
Microglia mediated oxidative stress leads to neurotoxicity.

Within microglia, ROS are associated with proliferation (7, 8), immune defense (9–11), and redox signaling (12). ROS-sensitive pathways mediate the activation of nuclear factor-kappa B (NFκB) and other transcription factors critical to the inflammatory response of microglia and other phagocytes (13). Not only do microglia use ROS as a defense against pathogens, but they activate pathways that allow microglia to respond to CNS injury. This rapid response of microglia, including migration to a site of injury, aids the clearance of debris and dead cells, preventing secondary cell death (14, 15). However, chronic activation of microglia and the overproduction of ROS is neurotoxic, precipitating oxidative stress which contributes to distinct secondary injury cascades, such as lipid peroxidation and oxidative histone phosphorylation (16) (Figure 1). Microglia are also a source of the highly toxic peroxynitrite (17). Activated microglia produce nitric oxide (NO), which rapidly reacts with superoxide due to its unpaired electron to form peroxynitrite (ONO_2_^–^), a reactive nitrogen species (RNS) toxic to both neurons and oligodendrocytes (17, 18).

Oxidative stress is an imbalance between generated ROS and innate antioxidants. Under oxidative stress, there is a greater probability that excessive ROS will react with lipids, nucleic acids, and proteins. These reactions induce lipid peroxidation and oxidation of proteins and DNA. Lipid peroxidation and DNA oxidation in particular can disrupt the plasma membrane and bring about DNA damage capable of inducing apoptosis after neurotrauma (19, 20). Thus, oxidative stress intensifies conditions of neurodegenerative disease and injury.

## Mechanisms of microglial reactive oxygen species generation

**FIGURE 2:**
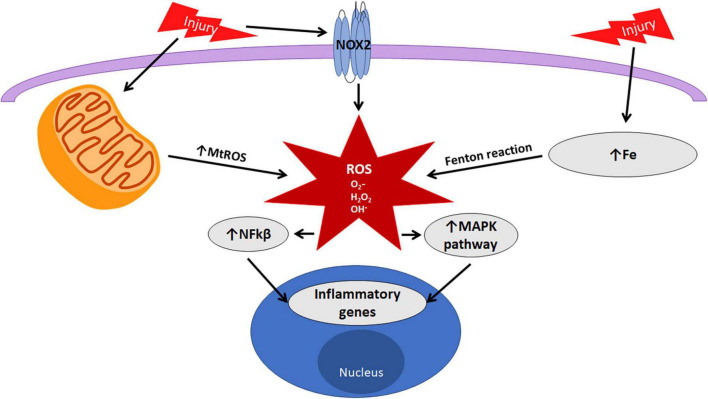
Oxidative stress in microglia. Microglia can produce reactive oxygen species (ROS) through NOX2, mitochondrial oxidative phosphorylation, and the Fenton reaction with iron. ROS act as signaling molecules to mediate pathways pivotal in microglial functions.

The production of ROS originating from multiple intracellular mechanisms must be strictly regulated to minimize excessive damage to the CNS. A number of these mechanisms are active in microglia. This review will focus on three of the most common inducers of ROS identified from microglia, nicotinamide adenine dinucleotide phosphate (NADPH) oxidase, iron, and mitochondria (Figure 2).

### Nicotinamide adenine dinucleotide phosphate oxidase

**FIGURE 3:**
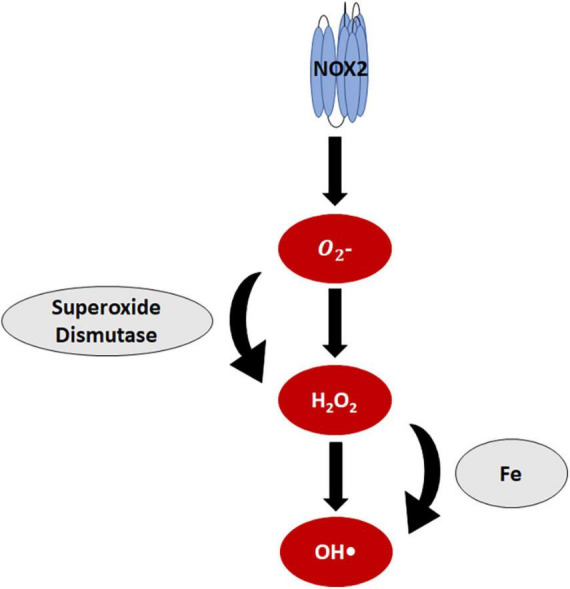
Reactive oxygen species in microglia. NOX2, the primary reactive oxygen species (ROS)-generating enzyme in microglia, produces superoxide. Superoxide dismutase then catalyzes the dismutation of superoxide to produce hydrogen peroxide. Iron reacts with hydrogen peroxide, generating hydroxyl radicals.

NADPH oxidase (NOX) is a primary ROS-generating enzyme that microglia express (21, 22). The NOX family consists of seven isoforms, most of which catalytically transfer one electron from NADPH to oxygen, producing superoxide. The antioxidant superoxide dismutase (SOD) can then dismutate superoxide to hydrogen peroxide (Figure 3), but imbalance in anti- and pro-oxidant components due to injury or disease can shift hydrogen peroxide concentrations and lead to oxidative stress.

Microglia express the isoforms NOX1, NOX2, and NOX4 (23, 24). Membrane bound NOX2 (gp91*^phox^*) is requisite for microglia-mediated neuroinflammation and is considerably increased after TBI and SCI (23, 25–27). NOX2 is activated in association with membrane bound p22*^phox^* and the translocation of cytosolic Rac2, p40*^phox^*, p47*^phox^*, and p67*^phox^*.

Plasma membrane bound NOX2 is the principal generator of extracellular superoxide, as evidenced by the finding that lipopolysaccharide (LPS)-stimulated microglial cultures derived from gp91*^phox–/–^* rodents fail to increase extracellular superoxide (28). Activation of NOX2 on the phagosome membrane releases superoxide into the lumen to be converted to other reactive species, facilitating anti-microbial function (29, 30). Studies have reported that microglial phagocytosis induces NOX-dependent superoxide production in response to cellular debris and IgG antibodies (31, 32).

NOX function is also necessary to activate phosphoinositide 3-kinase, MAP kinases, and NFκB, which upregulate microglial proinflammatory gene expression (33) and control LPS-induced phagocytosis (34). NOX-dependent NLRP3 inflammasome activity regulates the secretion of pro-inflammatory cytokines, which may mediate the neurodegenerative effects of microglial oxidative stress (35). Based upon these findings, NOXs present a significant source of ROS production while NOX pathways govern microglial functions, both of which influence neuroinflammation.

Microglial activation and subsequent neurotoxicity is in many cases NOX-dependent (28, 36). Other research suggests that NOX activity alone may not cause significant neuron death, but rather requires additional cellular mechanisms such as iNOS (37). Microglial NOX activity also contributes to the breakdown of the blood brain barrier (BBB). Astrocyte and endothelial cell death increases after oxygen-glucose deprivation/reperfusion when co-culturing with microglia, an effect prevented with NOX inhibition (38).

NOX2 has been shown to be particularly responsive to injury in the CNS, including TBI and SCI, with a rapid upregulation and maximal expression within the first 7 days after injury, and chronic expression through at least 1 month (23, 24). NOX2 activity is induced by stimulation of the toll-like receptors and requires induction of the NFκB and p38 MAPK signal transduction pathways (39). NOX2 and 4 have both also been implicated in post-SCI neuropathic pain development (40, 41).

A variety of therapeutics targeting ROS and the primary inducers of ROS in microglia have been developed, and a summary of some of these therapeutic approaches is presented in Table 1. Inhibition of microglial NOX attenuates pro-inflammatory gene expression, ROS, and oxidative stress (42–44). Reduced or indirectly inhibited function of NOX explains the anti-inflammatory effects of other neuroprotective treatments, including antioxidants and non-steroidal anti-inflammatory drugs (45–47). Although NOX2 is not the sole generator of ROS within activated microglia, it presents a possible therapeutic target to reduce oxidative stress and neuroinflammation after neurotrauma. In further sections, details of NOX therapy will be discussed.

**TABLE 1:** Therapeutics targeting microglial reactive oxygen species (ROS).

Model	Species	Therapy	Outcome	References
Microglia cell culture; Midbrain cell culture	Rat	Diphenyliodinium	Reduced microglial ROS, pro-inflammatory cytokines and NO	(42)
Intracerebroventricular LPS injection	Mouse	Apocynin	Change in microglial activation state toward anti-inflammatory	(43)
Microglia cell culture	Rat	Melatonin	Inhibition of p47 phosphorylation, reduction of ROS	(45)
Microglia cell culture	Mouse	Mdivi-1 (mitochondrial division inhibitor)	Normalization of mitochondrial membrane potential, reduced ROS and cytokines.	(66)
Microglia cell culture	Rat	Gp91ds-tat	Reduced cytokines, reduced ROS	(75)
Microglia cell culture	Mouse	Milk-fat globule-Epidermal growth factor – factor 8 (Nrf stimulant)	Reduced cytokines, reduced ROS	(109)
Alzheimer model; microglia cell culture	Mouse	Ibuprofen	Reduced oxidative stress markers, reduced NOX assembly in microglia	(46)
TBI (CCI)	Mouse	Apocynin	Improved sensorimotor function, reduced lesion volume, reduced NO.	(47)
TBI (CCI)	Mouse	Apocynin	Reduced microglial activation, oxidative stress, and beta-amyloid.	(44)
TBI (CCI)	Mouse	Microglia depletion	Cognitive function improved, reduced lesion volume, reduced NOX2 expression	(98)
TBI (CCI)	Mouse	NOX2 KO	Reduced lesion volume, reduced apoptosis, reduced oxidative damage	(16)
TBI (CCI)	Mouse	NOX2 KO, gp91ds-tat	Improved motor function, reduced inflammation, reduced microglial inflammation	(118)
TBI (CCI)	Mouse	NOX2 KO, Apocynin	Reduced oxidative damage, reduced lesion volume, reduced inflammation.	(117)
TBI (CCI)	Mouse	Gp91ds-tat	Reduced edema, reduced cell death	(44)
TBI (CCI)	Mouse	NOX2 KO; apocynin & tBHQ	Improved locomotor function, smaller lesion volume.	(106)
TBI (weight drop)	Rat	Apocynin	Reduced microglial activation, BBB disruption, and neuronal loss	(116)
TBI (weight drop)	Mouse	tBHQ	Improved neurological function, decreased oxidative stress	(110)
TBI (weight drop)	Mouse	tBHQ	Reduced NFκB, reduced inflammation, reduced apoptosis	(111)
TBI (CCI)	Rat	tBHQ	Reduced microglia, reduced cytokines, reduced astrocytes	(112)
TBI (weight drop)	Rat	MitoQ	Reduced pathology	(125)
TBI (CCI)	Rat	Ubiquinol	Reduced apoptosis	(126)
TBI (weight drop)	Mouse	MitoQ	Improved function, reduced neuronal apoptosis, increased Nrf2	(127)
Intracerebral hemorrhage	Mouse	MitoQ	Anti-inflammatory	(58)
TBI (weight drop)	Mouse	SS-31	Decreased ROS and oxidative damage, improved sensorimotor function	(131)
TBI (CCI)	Rat	Deferoxamine	Reduced histopathology, reduced function impairment	(135–138)
TBI (weight drop)	Rat	Minocycline	Improved neurological and motor function	(140)
TBI (weight drop)	Mouse	Minocycline	Reduced inflammation	(141)
TBI (CCI)	Mouse	HBED	Reduced microglia activation	(139)
TBI (weight drop)	Mouse	Reutaecarpine	Reduced microglial activation, neurological improvement, reduced oxidative damage	(114)
TBI (weight drop)	Mouse	Ramelteon	Reduced lesion volume, neurological improvement, NRF2 elevation	(115)
SCI (compression)	Rat	Methylprednisolone	Reduced oxidative damage, improved motor function	(152)
SCI (contusion)	Mouse	Iron chelator salicylaldehyde isonicotinoyl hydrazine	Reduced iron, limited motor function improvement	(85)
SCI (weight drop)	Rat	Deferoxamine	Mild improvement in motor function	(169)
SCI (contusion)	Rat	Deferoxamine	Motor function improvement	(170)
SCI (contusion)	Rat	Diphenyliodinium	Reduced oxidative stress	(24)
SCI (contusion)	Mouse	Gp91dst-tat	Functional recovery, reduced microglial ROS	(27)
SCI (contusion)	Mouse	Apocynin	Improved motor function	(159)
SCI (contusion)	Mouse	Gp91ds-tat	Improved motor function, reduced inflammation, reduced oxidative stress	(160)
SCI (contusion)	Mouse	NAC	Reduced microglial ROS, reduced microglial activation, reduced microglial mitochondria dysfunction	(164)
SCI (contusion)	Rat	Ketogenic diet	Improved motor function	(165)
SCI (contusion)	Mouse	HV1 knockout	Reduced NOX2 expression, ROS production and inflammation, improved motor function	(162, 163)
SCI (contusion)	Mouse	Microglial depletion	Reduced ROS, improved motor function	(158)
SCI (crush injury)	Mouse	Microglial depletion	Worsened motor function	(15)

### Mitochondria

Oxidative phosphorylation can generate mitochondrial ROS (mtROS) within the intermembrane space and matrix. As electrons are passed through the electron transport chain to form ATP, both complex I and complex III can release electrons to form superoxide. The rate of this ROS production normally remains low, and mitochondrial SOD reduces mtROS to form hydrogen peroxide, which glutathione peroxidase then converts to water (48). The inner mitochondrial membrane transporter, uncoupling protein 2 (UCP2), downregulates mtROS. Decreased expression of UCP2 increases ROS in other phagocytes to provide infection resistance (49). Activated microglia increase mtROS via downregulation of UCP2. In fact, the phasic down- or upregulation of UCP-2 modulates microglial phenotype, such that UCP2-silenced microglia exhibit a continuous pro-inflammatory phenotype (50).

In addition, the co-occurring increase in superoxide and nitric oxide in activated microglia heightens the probability of producing peroxynitrite, which inhibits the electron transport chain (51, 52). Experimental administration of electron transport chain inhibitors enhances mtROS production and pro-inflammatory microglial activation (53, 54).

Like NOX2-induced ROS, increased mtROS can be transported to the cytoplasm and regulate microglial pro-inflammatory gene expression via MAP kinases and NFκB (53, 55). MtROS also activate the NLRP3 inflammasome (56). These results indicate not only that mtROS govern microglial activation, but that mtROS present a positive feedback loop resulting in mitochondrial dysfunction and elevated microglia-mediated oxidative stress (Figure 2).

Mitochondrial functioning impacts the outcomes of neurotrauma. For example, chronic inflammation due to neurotrauma may cause cells to consume the antioxidant, glutathione, faster than it can be replenished. Reduced glutathione levels are associated with increased electron transport chain complex activity without increasing ATP production as demonstrated with *in vivo* injections of LPS (57). Conversely, increased mitochondrial antioxidants can effectively reduce ROS and attenuate microglia-induced inflammation (58).

Mitochondrial metabolism is reduced at 1 h after SCI (59), and mitochondrial dysfunction is noted at 12 h post-injury in animal models, accompanied by increases in protein nitrosylation, lipid peroxidation and protein oxidation (60). However, it is important to note that increases in 3-nitrotyrosine (3NT), 4-hydroxynonenal (4HNE) and other oxidative stress markers have been noted prior to this 12 h time point, suggesting that while mitochondrial ROS certainly plays a role, additional sources of ROS are also at work. Similar alterations are observed in TBI, with acute disruptions in mitochondrial function that are associated with elevated ROS and oxidative stress (61, 62).

Intracellular mtROS production is also associated with mitochondrial fission in activated microglia (63, 64). LPS-stimulated microglia release mitochondria fragments that lead to an increase in astrocyte ROS and mitochondrial fragmentation (65), propagating oxidative stress.

To counter unfavorable mitochondrial functioning, microglia treated with mitochondrial division inhibitor show normalized glycolysis and oxidative phosphorylation and reduced ROS (66). In addition, UCP2 overexpression is neuroprotective in brain injured mice and *in vitro* cortical neurons subjected to oxygen glucose deprivation (67). From these data, it is apparent that the level of mtROS determines mitochondrial activity and dysfunction in altering microglial function and oxidative stress.

### Iron

Iron is an essential cofactor and molecular component for many intracellular mechanisms of neurons and glia. Iron is necessary for the function of NOX, oxidative phosphorylation, and the citric acid cycle (68, 69). The iron-binding protein, ferritin, sequesters intracellular iron, preventing iron toxicity. Free iron that is unbound to ferritin impairs phagocytosis (70) and contributes to oxidative stress. The Fe^2+^ iron ion reacts with the hydrogen peroxide generated from oxidative respiration or NOX to produce a hydroxyl radical in a catalytic conversion known as the Fenton reaction (Figures 2, 3).

Iron combined with mitochondrial oxidative stress can cause microglia to undergo ferroptosis, an iron-dependent programmed cell death (71, 72), or induce ferroptosis in other cells via iron dysregulation or NO release (73, 74). Recent research from our laboratory demonstrated that iron induces ROS production in microglia and amplifies ROS of stimulated microglia (75). Both NOX2 and NOX4 inhibitors prevent the effects of iron treatment on microglia.

Not only does iron exacerbate oxidative stress, but inflammatory states and ROS alter iron levels. Under physiological conditions, microglial ferritin levels are associated with levels of free iron (76). Prior studies have found mixed results determining whether microglial ferritin expression is decreased (77) or increased (78) with oxidative stress and LPS activation. Divalent metal transporter-1 (DMT1) and transferrin receptor-mediated endocytosis transport Fe^2+^ and Fe^3+^, respectively, to regulate intracellular iron levels (79). Meanwhile, ferroportin is the primary iron exporter. Pro-inflammatory stimuli cause an increase in intracellular iron accumulation and induce an upregulation of DMT1 in neurons, astrocytes, and microglia (80). Additional investigations revealed that microglia increase iron influx in response to both pro- and anti-inflammatory cytokines (81). Microglia may alter substrate preference for transferrin-bound iron under pro-inflammatory states or non-transferrin-bound iron under anti-inflammatory states (79). While Urrutia et al. (80) found neither TNF-α, IL-6, nor LPS significantly altered ferroportin expression among microglia, Holland et al. (78) found that IFNγ reduced microglial ferroportin. Microglial iron uptake is initially neuroprotective by reducing free extracellular iron and ROS (82, 83); however, the resulting interactions between iron and oxidative stress mediators within microglia contribute to microglia-mediated neurotoxicity. Future studies are essential to evaluate whether microglial uptake of iron impacts ferroptosis among other CNS cell types.

Excessive iron accumulation may also contribute to post-injury oxidative damage. Liu et al. demonstrated that iron was a primary contributor to hydroxyl radical formation within 5 h after spinal cord contusion injury (84). Iron staining has shown that there is an increase in iron phagocytosis by microglia/macrophages in the lesion site through 14 days post-injury (85). Magnetic resonance imaging showed that iron deposits are present through 35 days post-injury, both in the lesion site and distant to it, in macrophages/microglia, astrocytes and oligodendrocytes (86). In addition to uptake within cells, diffuse staining with Prussian blue suggests deposition of iron in the extracellular matrix (86). Iron homeostatic proteins are upregulated after injury, including ceruloplasmin (CP), which is increased at 1 day and remains elevated through 21 days post-SCI in astrocytes and macrophages near the lesion site and ferritin protein, which is increased from 3 days post-injury (85).

## Targeting microglia-mediated oxidative stress as a therapy for traumatic brain injury

Traumatic brain injury is associated with microglial activation and oxidative stress. Oxidative damage post-TBI can impair neuronal functioning, disrupt the BBB, and potentiate cell death (87). Postmortem analysis on human TBI brains found elevated cortical expression of microglial NOX2 and increased 8-hydroxydeoxyguanosine (8-OHdG), a predominant form of ROS-induced oxidative damage to DNA (88). A rodent TBI model, using the controlled cortical impact (CCI) showed increased cortical 8-OHdG in rats within 15 min of the injury (89). Other markers of protein and lipid oxidation, 4HNE and 3NT, vary with injury models, with studies showing an increase by 3 h and resolving by 24 h after blast injury (90) and others peaking between 24-48 h after a CCI injury (91). 4HNE was observed in the brain within 6 h after blast exposure (92, 93). Human brain tissue collected within 91 h of a TBI also displayed prominent evidence of oxidative stress (94). These elevations in markers of oxidative stress were often associated with areas of microglial activation (90). Oxidative damage is not just an acute response, and has been noted through 42 days after blast exposure (95). After a CCI, mice show evidence of oxidative stress in neurons and microglia through 8 months after injury (96). However, despite temporal variations between injury models, oxidative stress is an established pathophysiological process in human and animal models of TBI.

This section will highlight recent research that examined therapies and pharmacological agents targeting microglia-mediated oxidative stress in experimental models of TBI.

### Microglial depletion or general anti-oxidant treatment in traumatic brain injury

Chronically activated microglia are observed in patients with a TBI up to 17 years after a single injury (97). Microglial depletion may be a mechanism to reduce chronic microglia mediated oxidative stress. Using microglial depletion, Henry et al. (98) showed, in a mouse CCI injury model, that depleting microglia during the chronic phase of TBI followed by repopulation resulted in marked improvements in downstream neurological dysfunction and reduced posttraumatic neurodegeneration. Coinciding with the behavioral and cognitive improvements, histological analysis revealed a significant reduction in the lesion volume and an increased density of neurons in the cortex and dentate gyrus. Transcriptional analysis documented various oxidative stress-related genes that were differentially expressed in the cortex 2 months post-injury, including a reduction of oxidative stress-inducing genes and an increase in antioxidant-related genes. Furthermore, repopulated microglia presented as less reactive with significantly more ramifications, less hypertrophy, and a reduction in the expression of NOX2 characteristic of a less inflammatory response. The results from this study implicate reactive microglia in oxidative stress, cognitive/behavioral dysfunction and neuropathology associated with TBI. However, microglial reduction as a therapeutic approach is limited, considering the necessity of microglia in normal physiology of the brain. Further work in this field is necessary to truly understand the overall contribution of microglia to TBI.

In addition to non-specific microglial depletion, non-microglial specific oxidant reduction has been tested as a therapeutic target. Nuclear erythroid 2-related factor 2 (Nrf2) is known to bind to the antioxidant response element to upregulate antioxidant gene expression and counter oxidative stress. Increasing evidence suggests Nrf2 also modulates the functions of both mitochondria and NOX2 (99, 100). Protein levels of Nrf2 are increased after weight drop and blast models of TBI in rats and mice (101, 102), peaking at 1 day post-injury in neurons and 7 days post-injury in microglia near the nucleus, which suggests active transcriptional activity (103). However, Nrf2 may be downregulated during chronic stages of TBI (104). Nrf2 KO mice had greater neurological impairment, increased lesion volume, and higher levels of oxidative stress markers (4HNE, 8-OHdG, and protein carbonyls) in response to a CCI injury (105, 106). Nrf2 KO mice also showed exacerbated neuroinflammation, 4HNE, and 3NT 1 day following a fluid percussion injury (107). *In vitro* studies have confirmed that Nrf2 suppresses the microglia pro-inflammatory phenotype (108, 109).

Oxidative stress and microglial activation post-TBI can be altered with Nrf2 activators, of which tertiary butylhydroquinone (tBHQ) is the most well studied. Three doses of tBHQ (50 mg/kg) prior to mouse weight drop TBI improved neurological function, decreased the oxidative stress marker malondialdehyde (MDA), and increased SOD activity (110). One week of a tBHQ-supplemented diet before a weight drop injury reduced NFκB, proinflammatory cytokines, and apoptotic cell death (111). TBHQ (25 mg/kg) given at 5 min or 2 h post-CCI followed by a second dose at 24 h improved motor functioning but had no effect on lesion volume. However, lesion volume was reduced by combining the treatment with apocynin (106). Daily administration of tBHQ (25 mg/kg) beginning 24 h post-CCI significantly reduced CD68 + /Iba1 + microglia immunostaining concurrent with attenuated proinflammatory cytokine levels in rats 3 days post-injury. Treatment also reduced astrocyte activation at 7 days post-injury and lesion volume at 28 days post-injury (112). The results of these studies suggest tBHQ activates Nrf2 to offset oxidative stress and provide neuroprotective effects after TBI. Unfortunately, Nrf2 affects a multitude of intracellular targets, making it difficult to pare down Nrf2-targeted treatments to a particular effector such as the mitochondria or NOX2. A limitation of tBHQ is its debated genotoxicity, but it is approved for human consumption in small concentrations (113).

More recently, additional therapeutics targeting Nrf2 have been identified that may also hold promise after TBI, although the specific cellular target remains unclear. In these studies, approaches including rutaecarpine and ramelteon, have been shown to reduce neuronal oxidative damage and microglial related inflammation via Nrf2 pathway activation after TBI in mice models (114, 115). Additional study to identify mechanism of action and cellular source is necessary to continue to refine therapies and translate to clinical trials.

### NOX2 knockout or inhibition in traumatic brain injury

NOX2 activity is involved in post-TBI neuropathology and oxidative stress damage (16, 44, 116). Rodent studies of TBI show elevated microglial NOX2 expression within days (16, 117) and sustained at 28 days (118) and 12 months post-injury (119). Activated microglia expressing NOX2 are present in the periphery of a lesion up to one year following an injury (119). To study the pathophysiological contributions of NOX2 in a TBI, multiple studies have used NOX2 knockout (KO) mice in a CCI model. Dohi et al. (16) found that 2 days post-injury, NOX2 KO mice had reduced lesion volume and peri-lesional apoptotic cells. This was associated with decreased oxidation products ethidine and 3NT in CD11b + microglia and infiltrating macrophages. Kumar et al. (118) extended these findings, showing a reduced lesion volume and neuronal cell death at 21 days post-injury. Injured NOX2 KO mice also demonstrated improved motor function at 14 and 21 days post-injury. Genomic analysis of inflammatory markers in the cortex 1-day post-injury revealed a marked reduction in proinflammatory (NOS2, TNFα, IL-6, IL-12b, IL-1β) and increased anti-inflammatory genes (IL-4Rα, SOCS3, and Ym1). Moreover, the number of Iba1 + microglia/macrophages expressing the pro-inflammatory-associated CD16/32 cell surface marker was diminished at 7 days post-injury. In a separate study, Wang et al. (117) corroborated the neuroprotective effects of knocking out the NOX2 enzyme, by showing that NOX2 KO TBI mice displayed less oxidative damage (shown by reduced 4HNE) as well as increased neuronal survival and reduced lesion volume at 4 and 7 days after injury. Furthermore, this was associated with significantly downregulated pro-inflammatory-associated genes (CD16, CD32, CD86, iNOS) and significantly upregulated anti-inflammatory-associated genes (CD206, Ym1). Interestingly, when microglia were isolated from KO and wild-type TBI mice, the microglia from the former were less pro-inflammatory and more anti-inflammatory compared to the latter, with cells expressing less pro-inflammatory associated CD86 and more anti-inflammatory associated CD206. Lastly, *in vitro* studies showed that healthy neurons co-cultured with microglia from injured NOX2 KO mice were healthier, displayed less neuronal apoptosis, and cytotoxicity compared to neurons co-cultures with microglia from injured wild-type mice. This suggests that the activation of the NOX2 receptor on microglia in part, mediates the neurotoxicity observed in the *in vivo* injury model.

A limitation of these studies is the NOX2 KO mouse is a complete knockout of the NOX2 gene and not microglia specific. Therefore, effects may be partially mediated by additional cell types. Given that neurons also express NOX2, some neuroprotective effects of NOX2 KO could be related to direct intracellular effects on neurons. Additionally, studies used markers that cannot distinguish microglia from macrophages, which could be contributing to results. In fact, Kumar et al. (118) determined that macrophages accounted for 25% of NOX2 expression post-CCI while microglia accounted for 8%. Further research is needed to investigate the contributions of microglial versus macrophage NOX2 following TBI.

Researchers have also tested the therapeutic potential of targeting the NOX2 enzyme with various pharmacological modulators. The specific peptide inhibitor, gp91ds-tat, has been utilized to target NOX2 in CCI injury models. Administration of gp91ds-tat (250 μg/mouse) 20 min prior to CCI reduced edema 1-day post-injury and cell death 4-days post-injury (44). Consistent with the aforementioned KO studies, administration of gp91ds-tat (5 mg/kg) at 24, 48, and 72 h post-CCI reduced CD16/32 expression in the cortex (120) and promoted an anti-inflammatory microglia phenotype, indicated by increased anti-inflammatory Arg1 and Ym1 expression in P2Y12 + /CD11b + microglia (118). Treatment was associated with improved performance in spatial working memory tests but had no improvement on fine motor function within the first week post-injury (118).

Apocynin is a medicinal compound isolated from the plant *Picrorhiza kurroa* and inhibits NOX activity in neutrophils and macrophages by preventing p47^phox^ and p67^phox^ subunit translocation (121). Choi et al. (116) showed that rats pre-treated with apocynin (100 mg/kg) fifteen min before a weight drop model of TBI had reduced microglial/macrophage activation in the CA1 region of the hippocampus 7 days after injury. Additionally, this was associated with reduced BBB dysfunction and fewer degenerating neurons in the hippocampus. Using a CCI mouse model of TBI, researchers found that administration of apocynin (4 mg/kg) 20 min before injury or 2 h post-injury significantly reduced immunostaining for oxidative stress markers 4HNE, 8-OHdG, and p-H2AX in the cortex and CA1 regions of hippocampus 2 days post-injury (44). The decrease in oxidative stress was followed by reduced microglia/macrophage activation in the cortex and CA1 region of the hippocampus 4 days post-injury. Another CCI mouse study found that apocynin (5 mg/kg) administered for 4 consecutive days prior and 1 day post-injury significantly decreased ROS (measured with hydroethidine) within CD11b + microglia/macrophages (117).

Although these results suggest apocynin has NOX-mediated anti-inflammatory effects on microglia, the effects of apocynin are not specific to a single NOX isoform and have off-target effects (122). For this reason, the favorable outcomes of apocynin treatment may indicate a benefit of general antioxidant treatments for TBI. In addition, global NOX2 or NOX inhibition may have negative immune suppressive effects. Reports of gastrointestinal disorders or dysfunction of neutrophils have been reported with NOX2 inhibition (123), which suggest that additional research is needed to better target NOX2 inhibitors to microglia or to the brain after injury.

### Targeting mitochondria in traumatic brain injury

Treatments aimed to alter the function of mitochondria or its electron transport chain may limit ROS production and suppress oxidative stress. Cortical mitochondria isolated post-CCI show phasic dysfunction, with significant oxidative damage beginning at 24 h, allowing for a larger therapeutic window for treatments (124). Antioxidant compounds present one treatment strategy to target mitochondrial dysfunction post-TBI. Here we will discuss just a few mitochondria-targeted antioxidants.

Coenzyme Q10 (CoQ10), or ubiquinone, is an essential part of the electron transport chain, but it also acts as an antioxidant. TBI outcomes have been assessed following treatments with CoQ10, its reduced form ubiquinol, and mitoquinone (MitoQ) which is composed of covalently bound CoQ10 and triphenylphosphonium ions. CoQ10 (10 mg/kg) administered immediately after a weight drop injury reduced MDA, generated a non-statistically significant increase in SOD, and attenuated TBI-induced histopathology (125). Ubiquinol (100 mg/kg), administered to rats 30 min before a CCI mitigated mitochondrial damage and reduced apoptosis (126). Administration of MitoQ (4 mg/kg & 8 mg/kg) 30 min after injury reduced neuronal apoptosis and improved neurobehavioral function at 1 and 3 days post-injury. MitoQ also reduced post-TBI oxidative stress, increasing the antioxidant activity of glutathione peroxidase and SOD, while decreasing MDA. MitoQ also increased Nrf2 activity and upregulated downstream proteins of Nrf2 signaling (127). Although the above studies did not confirm the association between treatments and microglial activation, MitoQ did have neuroprotective effects and anti-inflammatory effects on microglia *in vivo* after intracerebral hemorrhage (58). Despite these results, separate studies have found that MitoQ can cause mitochondrial damage due to increased inner membrane permeability (128) and increase mitochondrial ROS driven by complex I activity (129). Further research is necessary to corroborate the effects of CoQ10-based treatments on microglial functions and microglia-mediated oxidative stress.

SS-31, also known as elamipretide or bendavia, is a synthetic antioxidant peptide that binds to cardiolipin of the inner mitochondrial membrane where it inhibits cytochrome c peroxidase activity, inhibits mitochondrial permeability, and reduces mitochondrial ROS (130). SS-31 (5 mg/kg & 10 mg/kg) administered to mice 30 min after a weight drop injury decreased ROS, 8-OHdG, MDA, and increased SOD (131). Treatment also improved sensorimotor functioning and reduced apoptosis as well as intracellular iron loads. SS-31 (5 mg/kg) given to mice 30 min before an intracerebral LPS injection followed by three daily doses attenuated ROS, MDA, and pro-inflammatory cytokine levels while also increasing SOD in the hippocampus (132). LPS-stimulated microglia pre-treated with SS-31 showed attenuated mitochondrial fragmentation, iNOS and COX2 expression, and ROS production (133). Mice subject to brain ischemia/reperfusion immediately followed by SS-31 treatment (5 mg/kg) had reduced microglia/macrophage activation in the injured hemisphere (134). Safety or efficacy of SS-31 for the treatment of neurotrauma has not been confirmed in humans, but clinical trials may be forthcoming.

### Targeting iron in traumatic brain injury

Although iron is essential to the healthy brain, iron overload can exacerbate oxidative stress. Accordingly, iron chelation therapy has been tested as a treatment of TBI. Administration of the iron chelator, deferoxamine has demonstrated some efficacy in improving histological and functional outcomes of CCI, lateral fluid percussion, and weight drop models of TBI on mice and rats, but investigations of deferoxamine’s effects on oxidative stress and microglial activation remains incomplete (135–138). Mixed findings are likely a result of deferoxamine being limited to access the CNS in areas of BBB damage.

Hydroxybenzyl ethylenediamine (HBED) is an iron chelator which can cross the BBB. HBED (100 mg/kg) followed with bidaily doses (50 mg/kg) for 3 days post-CCI had a neuroprotective effect with reduced microglial activation but had varying results on functional outcomes (139).

Minocycline, an antibiotic that has iron chelation properties and can also stabilize mitochondrial function, improved neurological and functional outcomes of a weight drop model of TBI on rats using multiple dose concentrations (140). A separate weight drop study using mice found minocycline (45 & 90 mg/kg) reduced inflammation but had no effect on the glutathione reduction ratio as a marker of oxidative stress (141). Minocycline reduced microglial activation in human TBI patients but also increased neurofilament light, a marker of neurodegeneration in humans (142).

In humans, iron chelators used for the treatment of iron overload syndromes have painful and potentially neurotoxic side effects (143). After lateral fluid percussion in rats, long-term term iron deposits and chronically activated microglia are associated with BBB leakage, which suggests treatments aimed at treating BBB dysfunction would be a more efficient target for both iron and overall neuroinflammation (144). In conclusion, although iron chelation may attenuate microglia-mediated oxidative stress, iron-targeted treatments may not be as effective or protective as other oxidative stress targets involved with TBI.

## Targeting microglia-mediated oxidative stress as a therapy for spinal cord injury

Injury to the spinal cord results in acute and long-lasting oxidative stress at both the lesion site and surrounding tissue. Interestingly, measurements of local oxidative stress have shown higher levels of ROS, lipid peroxidation and mitochondrial DNA oxidation in spinal cord after SCI than in cortical tissue after brain injury (145), suggesting that spinal cord tissue may be particularly sensitive to oxidative damage or contain cells that are primed toward a shift to oxidative stress. In blood samples from patients with cervical SCI, markers of oxidative stress were elevated acutely and up to 7 days after injury, while antioxidant levels were reduced (146). In rats with contusion SCI, markers of oxidative stress, including 3NT and 4HNE, were elevated by 3 h post-injury (147). The expression of these markers expanded throughout the tissue, into both gray and white matter, through 72 h post-injury. This expansion of expression began to decline by 2 weeks post-injury. In rabbits with spinal cord ischemia, markers of DNA oxidation (8-OHdG) were found to be elevated by 8 h after reperfusion, and peaked at 24 h (148). In a study of multiple models of SCI, contusion, dislocation and distraction injuries in rats were all found to increase oxidative stress markers within 3 h after injury, particularly in neurons, with slight differences in foci (149). Peroxynitrite after SCI shows an increase by 1 h that was sustained through 1 week, with a peak at 24 h (150).

Oxidative stress is not just an acute response to CNS injury, however. Analysis of SCI tissue at 1 month after SCI showed reductions in antioxidants (glutathione, alpha tocopherol) and increases in oxidative stress markers (epi-prostaglandin F2 alpha); these alterations remained present through 12 months after injury (151).

Treatment to reduce oxidative stress after SCI has been varied. One of the first treatments approved for SCI treatment, methylprednisolone, was found to significantly reduce oxidative damage in the injured spinal cord (152). However, methylprednisolone was found to impair systemic inflammatory responses and have other negative systemic effects. Therapies that have focused on directly scavenging oxidants after SCI have also proven somewhat successful in preclinical trials (153), but therapeutic windows for these approaches seem to be short and focused on the acute burst of ROS production (154). Therefore, treatments focusing on spinal cord specific oxidative stress may be more beneficial.

### Microglial depletion in spinal cord injury

Directly reducing microglia in the injured spinal cord by microglial depletion has been met with conflicting results and appears to be heavily dependent on timing of the depletion initiation. Acute depletion or depletion prior to injury using PLX3397 or genetic manipulation was found to reduce functional recovery and impair neuronal survival after SCI (15, 155, 156). A recent study demonstrated that deletion of microglia prior to SCI led to a disruption in the glial scar (157). These data suggest that acute activity of microglia is essential to injury recovery.

However, delayed depletion using PLX5622 reduced microglial ROS and improved outcomes (158). This study demonstrated that PLX5622 administration starting at 1 day post-injury, in which microglia were depleted by 7 days post-injury, significantly improved motor function of mice after SCI and reduced long-term expression of inflammatory and apoptosis markers. Therefore, one can conclude that microglia within the injured spinal cord play a complicated and nuanced role that can not be overlooked or eliminated for therapeutic efficacy. Targeting specific microglial functions, such as ROS production, may then optimally improve outcomes.

### NOX2 knockout or inhibition in spinal cord injury

NOX2 inhibition has been shown to reduce oxidative stress and improve functional recovery after SCI (24, 27). Using the non-specific NOX inhibitor apocynin, Zhang et al. (159) showed that acute systemic NOX inhibition improved functional recovery in aged, but not young, female mice, suggesting an age-related impact of treatment. These functional improvements were associated with significant reductions in macrophage invasion and macrophage-related ROS production. Interestingly, we have shown that in young male mice, NOX inhibition with gp91ds-tat acute intrathecal infusion did significantly improve functional outcomes in young mice (160), accompanied by significant reductions in local ROS markers and microglial numbers. This difference suggests that location (systemic vs. intrathecal) of treatment may also have a marked impact on treatment efficacy. Alternatively, sex may play a role, as systemic administration of gp91ds-tat significantly improved motor function in young adult male mice after a T10 contusion injury (27); this beneficial effect was associated with a significant reduction in ROS production in Iba1 + cells, although whether these cells were microglia or macrophages was not determined. The therapeutic approach to targeting NOX may also be the reason – apocynin is a non-specific approach; gp91ds-tat, which directly blocks NOX2 activity by binding the p47^phox^ cytosolic component instead of allowing for p47^phox^ to bind to the enzymatic core gp91^phox^, may be a more efficient method for inhibition (161).

In addition, targeting of the microglial proton channel HV1, which has been linked to NOX2 generated ROS, for genetic depletion has been shown to reduce NOX2 expression, ROS production and neuroinflammatory gene expression and improve motor function after SCI (162, 163).

### Targeting mitochondria in spinal cord injury

Therapies targeting mitochondrial ROS have also been shown to reduce SCI oxidative stress and improve functional outcomes. Administration of n-acetylcysteine (NAC) has been shown to reduce ROS production by microglia *in vitro* and in SCI, accompanied by reduced microglial activation and reduced microglial mitochondrial dysfunction (164). Ketogenic diets, in which β-hydroxybutyrate metabolites are presumed to be able to bypass damaged metabolic pathways and enter the TCA cycle in mitochondria to assist in production of ATP, have been shown to improve functional outcomes after SCI (165). The ketogenic diet was also found to increase antioxidant availability and reduce cytokine production. Administration of minocycline, which is reported to stabilize mitochondria and inhibit the release of cytochrome c, has also been reported to reduce oxidative stress and improve outcomes after SCI (166–168).

### Targeting iron in spinal cord injury

Iron chelation therapy has proven effective in reducing oxidative stress in the injured spinal cord (85). Systemic administration of the lipophilic iron chelator salicylaldehyde isonicotinoyl hydrazine twice a week starting 1 h after SCI significantly reduced iron presence in the injured cord (85). However, despite this reduction, improvement in motor function was limited, with a significant but only 1 point improvement in the Basso Mouse Scale by 42 days post-injury. Deferoxamine, another iron chelator, administration after SCI was found to have similar results – depressing iron accumulation and inducing mild improvements in motor function (169). Increasing the frequency of treatment with deferoxamine may improve outcome – daily administration of deferoxamine starting 30 min prior to SCI was shown to significantly reduce iron load in the injured cord and significantly improve motor scores by at least 2 points (170). Daily administration after spinal cord compression was also found to improve recovery and induce vascularization (171). A recent study showed that deferoxamine administration after SCI improved Basso, Beattie and Bresnahan scale locomotor scores and reduced iron accumulation in the brain as well, reducing oxidative stress in the motor cortex (73). More importantly, deferoxamine administration has been shown to specifically act on microglia to reduce oxidative stress in an SCI-pain model (172). However, other studies have shown that oral administration of iron chelators, such as deferasirox, have moderate to no effect on motor function after SCI, and that high doses can be toxic (173). Therefore, additional research, particularly focused on non-systemic administration routes, is needed.

## Conclusion

It is important to note that much of the early work in characterizing oxidative stress temporo-spatial dynamics was performed in female animals; the contribution of sex-differences in oxidative stress responses is not well understood. Sex has been shown to play a significant role in several post-injury inflammatory responses, including cytokine release (174), inflammasome formation (175), and T and B cell responses (176). In SCI, recent work by Stewart et al. demonstrated that while male mice demonstrate an elevated number of microglia in the injured spinal cord vs. females, female mice showed more NOX2 gene expression than males (177). These data suggest that oxidative stress may differ substantially between the sexes. Further work is warranted to identify how these variables affect ROS production.

Targeting of microglia mediated oxidative stress enhances behavioral and functional outcomes after both SCI and TBI and shows promise as a possible clinical therapy. While many of the studies detailed above do not specifically target just microglia, the complementary microglial depletion work demonstrates several of the same effects, suggesting that microglia may be a key target of global antioxidant therapies. The current research identifies Iba1 + cells as major ROS producers, but more studies are needed to identify the differential effects of macrophages compared with microglia. It is clear that additional research is needed to understand the best circumstances for beneficial outcomes when inhibiting microglial ROS production, including NOX, iron or mitochondrial dysfunction. Longitudinal studies are needed to identify the impact of therapies at longer time points to ensure short term improvements are not being traded for negative long-term consequences. This work would be a necessary foundation to support the expansion into clinical trials. Resolving or understanding the mechanisms that drive mixed results is integral to moving therapies into human use.

To date, several therapeutics have been met with mixed results. More research is needed to identify how to appropriately target ROS sources to reduce post-injury oxidative stress, and this remains a rich area for study and therapeutic targeting.

## Author contributions

KB designed the overview of the manuscript and wrote the spinal cord injury section. AS researched and wrote the introduction and TBI section. MS researched and wrote the TBI section. DH designed the figures. SC wrote the abstract and conclusions. All authors contributed to the article and approved the submitted version.
